# Salivary interleukin-17A and interferon-γ levels are elevated in children with food allergies in China

**DOI:** 10.3389/fimmu.2023.1232187

**Published:** 2023-11-27

**Authors:** Yan Yin, Shengrong Ouyang, Qin Li, Yuyang Du, Shiqiu Xiong, Min Zhang, Wei Wang, Ting Zhang, Chuanhe Liu, Ying Gao

**Affiliations:** ^1^ Department of Integrated Early Childhood Development, Capital Institute of Pediatrics, Beijing, China; ^2^ Department of Biochemistry and Immunology, Capital Institute of Pediatrics, Beijing, China; ^3^ Environmental Standards Institute, Chinese Research Academy of Environmental Sciences, Beijing, China; ^4^ Department of Allergy, Affiliated Children’s Hospital of Capital Institute of Pediatrics, Beijing, China; ^5^ Department of Dermatology, Affiliated Children’s Hospital of Capital Institute of Pediatrics, Beijing, China

**Keywords:** food allergies, IL-17, IFN-γ, saliva, children

## Abstract

**Introduction:**

Food allergies have a substantial impact on patient health, but their mechanisms are poorly understood, and strategies for diagnosing, preventing, and treating food allergies are not optimal. This study explored the levels of and relationship between IL-17A and IFN-γ in the saliva of children with food allergies, which will form the basis for further mechanistic discoveries as well as prevention and treatment measures for food allergies.

**Methods:**

A case–control study with 1:1 matching was designed. Based on the inclusion criteria, 20 case–control pairs were selected from patients at the Skin and Allergy Clinic and children of employees. IL-17A and IFN-γ levels in saliva were measured with a Luminex 200 instrument. A general linear model was used to analyze whether the salivary IL-17A and IFN-γ levels in the food allergy group differed from those in the control group.

**Results:**

The general linear model showed a significant main effect of group (allergy vs. healthy) on the levels of IL-17A and IFN-γ. The mean IL-17A level (0.97 ± 0.09 pg/ml) in the food allergy group was higher than that in the healthy group (0.69 ± 0.09 pg/ml). The mean IFN-γ level (3.0 ± 0.43 pg/ml) in the food allergy group was significantly higher than that in the healthy group (1.38 ± 0.43 pg/ml). IL-17A levels were significantly positively related to IFN-γ levels in children with food allergies (r=0.79) and in healthy children (r=0.98).

**Discussion:**

The salivary IL-17A and IFN-γ levels in children with food allergies were higher than those in healthy children. This finding provides a basis for research on new methods of diagnosing food allergies and measuring the effectiveness of treatment.

## 1 Introduction

Food allergy (FA) is an adverse health effect characterized by an immune response that occurs reproducibly after exposure to a given food (1). FA can be divided into three categories: IgE mediated, IgE dependent and IgE independent pathway mediated (mixed), and non IgE mediated (2). Due to the release of mediators triggered by the binding of IgE antibodies to mast cells and basophils, IgE-mediated FA is characterized by immediate clinical manifestations. Non-IgE-mediated FA is an inflammatory response driven by T cells (3).

The various cell types involved in the occurrence and development of FA produce different cytokines and regulate different functions. The cytokines produced by Th2 cells, such as IL-4, IL-5, and IL-13, induce B cells to differentiate into plasma cells that produce IgE (4). Specific IgE antibodies induce degranulation of mast cells and release mediators such as cytokines, leading to allergic symptoms (5). T regulatory cells (Tregs) produce inhibitory cytokines such as IL-10 and TGF-β and play an important role in maintaining food tolerance and preventing the occurrence of FA (6).

Recently, functional research on Th17 has revealed the complexity of Th1/Th2 imbalance in FA, broadening the understanding of the pathogenesis of allergies (7, 8).Th17 cells mainly exist on the surface of the intestinal mucosa, which helps to maintain intestinal homeostasis (9, 10). A decisive feature of Th17 cells is their phenotypic flexibility, which allows them to easily undergo phenotypic changes based on the local environment. Research has found simultaneous expression of IL-17 and IFN-γ at the inflammatory site of Th17 cells, which are called Th17/Th1 cells (11, 12). An increasing number of studies have found that Th17/Th1 cells play a crucial role in the pathogenesis of many other intestinal immune diseases (13, 14).

These two important cytokines IL-17A and IFN-γ, also play important roles in allergic diseases. IL-17A can regulate various cytokines and induce allergen-specific Th2 cell activation, eosinophil and neutrophil accumulation, and serum IgE production (15). Previous studies have found that due to its key role in barrier immunity and its synergistic effects with other cytokines, such as TNF-α, IFN-γ, and IL-1, IL-17 plays an important role in many skin diseases (16), allergic diseases such as allergic rhinitis (17) and asthma (18), and inflammatory bowel disease (19, 20). IFN-γ, a pleiotropic cytokine, is a major proinflammatory cytokine that regulates CD8+ T-cell proliferation after antigen exposure (21) and affects the maturation, differentiation and antigen processing and presentation of dendritic cells (DCs), which promote B-cell division during the early proliferative response following primary antigen exposure (22). Given its crucial role in modulating innate and adaptive immunity, it is not surprising that IFN-γ regulates the development of allergic diseases (23), autoimmune diseases such as systemic lupus erythematosus (SLE) (24) and endothelial system diseases (25).

FA is mainly caused by the entry of food allergens into the gastrointestinal tract. However, few studies have directly evaluated the relationship between the immune environment of the human gastrointestinal mucosa and FA. The oral cavity is the starting point of the gastrointestinal mucosal system, containing antigen-presenting cells, lymphocytes, and related lymphatic structures (26), and saliva, as an important part of the oral mucosal environment (27), serves as a rich source of information for studying the relationship between the gastrointestinal mucosal system environment and FA (27). Pajno GB et al. (28) reported that although sublingual immunotherapy (SLIT) and epidermal immunotherapy (EPIT) have fewer side effects (allergic reactions and eosinophilic esophagitis) than oral immunotherapy (OIT), the desensitization effect of OIT was better than that of the first two.

Our study analyzed the levels of IL-17A and IFN-γ in the saliva of children with FA using a case–control study with age- and sex-matched groups to enhance FA mechanism research and highlight the need for early screening and evaluation of immunotherapy effects on FA.

## 2 Materials and methods

### 2.1 Cases and controls

The study protocol was approved by the Ethics Committee of the Capital Institute of Pediatrics (Approval No. SHERLLM2022017). The guardians of the study subjects read and signed an informed consent form for inclusion in the study. Written informed consent was obtained from the individuals’ legal guardian for the publication of any potentially identifiable images or data included in this article. A total of 20 pairs of children were included. Each pair of children contained one child with FAs and a healthy child.

Subjects with FA were recruited from patients who were admitted to the Allergy Department and Dermatology Department of the Capital Institute of Pediatrics (Beijing, China) from September 15, 2022, to March 31, 2023. Healthy controls were recruited from the children of employees. This study employed a 1:1 matched design, with the participants being matched for sex and age.

The diagnostic criteria for FA in children were as follows: 1) a history of illness and exposure to allergenic food, such as eggs, milk, or shrimp; 2) symptoms of FA, consisting of oral contact urticaria (i.e., immediate swelling and itching of the oral mucosa), skin symptoms related to FA (such as itching or redness), or suspected gastrointestinal symptoms related to FA (such as diarrhea, bloody stools, vomiting, abdominal pain, bloating, or reflux) after contact with allergenic foods; and 3) positive skin prick test (SPT) results or food-specific IgE antibodies. Individuals who met the first two criteria underwent the SPT or IgE testing with the consent of their guardians, and those who had a positive result on either test were included in the FA group.

The SPT was completed by trained medical staff. Skin pricks were made on the palmar side of the left forearm. The substances applied in the SPT included the following: (1) histamine (positive control solution); (2) physiological saline (negative control solution); and (3) egg, milk, fish and shrimp. All of the reagents in the SPT were from Xiehe Xinhualian Pharmaceutical Co., Ltd. The SPT was carried out according to the method recommended by expert consensus (29).

In the present study, the SPT results were judged based on a combination of European standards, namely, that a positive test was indicated when the largest diameter of the wheal of each particular test was >3 mm (30), and the domestic expert consensus on skin prick experiments (2020) (29). The criterion for judging skin pricks by the domestic expert consensus (31) was the ratio of the wheal area of the allergen to that of histamine (positive control solution) as follows: (-), no reaction or the same as the negative control; (+), more than 1/4 of the area of the histamine wheal (positive control solution); (++), at least 1/2 the area of the positive control wheal; (+++), same area as the positive control wheal; (++++), more than 2 times the area of the positive control wheal. Only participants who met the two criteria were included as FA subjects. Serum levels of food-specific IgE antibodies and corresponding reagents for the specific IgEs were measured with a Thermo Scientific Phadia 250 instrument according to the standard operating procedures for the machine and reagents.

The levels of specific IgEs were scored as follows: 0: less than 0.35 IU/ml; 1: 0.35-0.70 IU/ml; 2: 0.70-3.5 IU/ml; 3: 3.5-17.5 IU/ml; 4: 17.5-50 IU/ml; 5: 50-100 IU/ml; and 6: >100 IU/ml.

The healthy controls were recruited from the children of employees at the Capital Institute of Pediatrics and matched with cases based on age and sex. The inclusion criteria for the control children were as follows: 1) the children had no previous or current history of FA; 2) based on physical examination of the children and questions posed to the guardians by members of the research group, the children did not suffer from any systemic disease at the time of screening or in the past; and 3) the children were not suffering from infectious diseases, such as cold or flu.

### 2.2 Saliva collection

Saliva specimens (1 ml) were collected by the doctor or guardian after the children drank water or rinsed their mouths for 15 minutes. For sample collection, the older children spat saliva into an Eppendorf tube on their own under the supervision of a doctor. For younger children who would not spit saliva into the tubes on their own, the doctor or guardian used a small, sterilized pipette (capacity 3 ml) to collect saliva by aspiration. Each saliva sample was stored at -80°C until the day of cytokine testing.

### 2.3 IL-17A and IFN-γ detection

**Figure 1:**
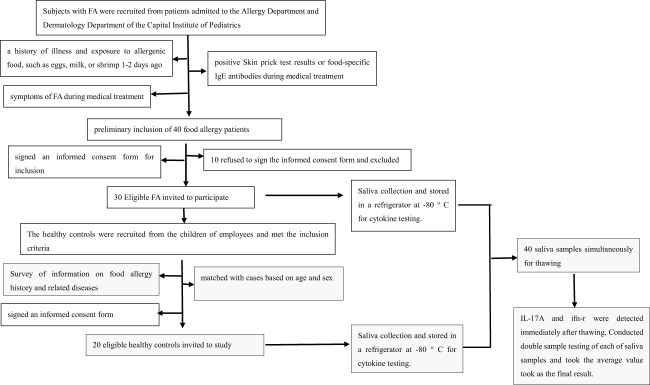
Flow diagram of the method.

On the day of detecting IL-17A and IFN-γ, all 40 saliva samples were simultaneously removed from the -80°C refrigerator to the refrigerator compartment for thawing. IL-17A and IFN-γ were analyzed with a Luminex 200 instrument at the Experimental Center of Capital Institute of Pediatrics, and the reagent was obtained from the MILLIPLEX Cytokine Detection Kit (product number HCYTOMAG-60K); the analysis was performed according to the instructions for the machine and detection kit. Double well tests on each sample were conducted, and the final test results of IL-17A and IFN-γ were taken as the average of the two test results. Flow diagram of the method was showed in **Figure 1**.

### 2.4 Statistical analysis

All analyses were performed with SPSS statistical software version 20.0 (SPSS, Inc.). Descriptive statistics were used to describe the characteristics of the study population, including the number and percent of participants per sex or age category. This study used a case–control design, with participants matched 1:1 for sex and age, to explore whether the levels of IL-17A and IFN-γ in saliva differed between the FA group and the healthy group and the difference in the concentrations of IL-17A and IFN-γ between children with FA and healthy children. A general linear model was used with IL-17A and IFN-γ as dependent variables and group (FA or healthy) and pair (each of the 20 matched pairs) as fixed factors. Pearson correlation analysis was used to analyze the linear correlation between IL-17A and IFN-γ. A p value of < 0.05 indicated statistical significance.

## 3 Results

### 3.1 Participant characteristics

This study included 20 pairs, each consisting of a child with FA and a healthy control. There were 10 pairs of boys and 10 pairs of girls, ranging in age from 6 months to 13 years. The sex and age distributions of the participants are shown in **Table 1**.

**Table 1:** Characteristics of the research subjects.

		N	Percent(%)	
Sex				
	boy	20	50	
	girl	20	50	
Age in months				
	6	2	5.0	
	14	2	5.0	
	22	2	5.0	
	30	2	5.0	
	36	2	5.0	
	41	3	7.5	
	42	4	10.0	
	49	2	5.0	
	50	6	15.0	
	60	2	5.0	
	61	2	5.0	
	62	2	5.0	
	64	2	5.0	
	97	4	10.0	
	144	2	2.5
	156	2	5.0	

Among the children with FA included in the study, two had a history of fish and shrimp allergies, four had a history of egg and milk allergies, and the remaining 14 had a history of egg allergies. Among these children, 16 had allergic symptoms such as rash, one had vomiting and diarrhea, two had urticaria, and one had rash and asthma. Fourteen children underwent the SPT, with 10 children receiving (++) results and four children receiving (+++) results. Six children underwent specific IgE testing, with results ranging from 1.50 to 35.0 IU/ml.

### 3.2 Distribution of cytokine levels between children with FAs and healthy controls

**Figure 2:**
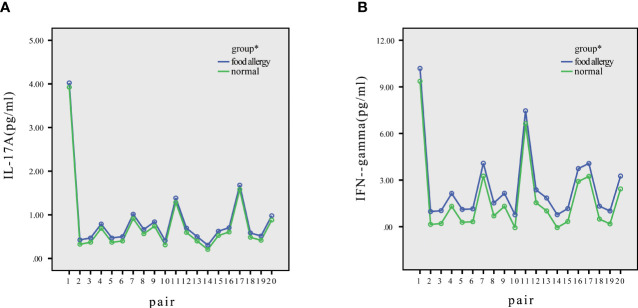
Distribution of IL-17A and IFN-γ in normal and allergic children. **(A)** The IL-17A levels of every pair were higher in allergic children than in normal children. **(B)** The IFN-γ levels of every pair were higher in allergic children than in normal children. *P<0.05.

Within all matched pairs of children, the concentrations of IL-17A and IFN-γ were higher in the children with FAs than in healthy children, as shown in **Figure 2**.

The distributions of IL-17A and IFN-γ significantly differed between healthy controls and children with FAs, as shown by significant main effects (p < 0.05) in the general linear model (**Table 2**).

**Table 2:** Levels of IL-17A and IFN-γ in the saliva of matched healthy and allergic children: main effects in the general linear model.

		Type III sum of squares	df	Mean square	F	p
Group	IL-17A (pg/ml)	0.78	1	0.78	5.32	<0.05
	IFN-γ (pg/ml)	26.07	1	26.07	7.06	<0.05

Moreover, the marginal mean level of IL-17A (0.97 ± 0.09 pg/ml) in the FA group was higher than that in the healthy group (0.69 ± 0.09 pg/ml). The marginal mean IFN-γ level (3.0 ± 0.43 pg/ml) in the FA group was higher than that in the healthy group (1.38 ± 0.43 pg/ml) (p<0.05). The power values of the tests for the differences in IL-17A and IFN-γ between the allergic and healthy groups were 71.3% and 59%, respectively, as shown in **Table 3**.

**Table 3:** Comparison of IL-17A and IFN-γ between children with FA and healthy controls by general linear model analysis.

	FA group	Healthy	F	p	Observed power
IFN-γ (pg/ml)	0.97 ± 0.09	0.69 ± 0.09	7.06	<0.05	71.3%
IL-17A (pg/ml)	3.00 ± 0.43	1.38 ± 0.43	5.32	<0.05	59%

### 3.3 Correlation of IL-17A and IFN-γ

**Figure 3:**
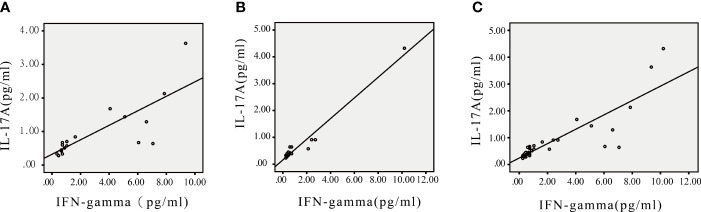
Scatter plots of IL-17A and IFN-γ in children with FA, healthy children and all participants. **(A)** IL-17A was correlated with IFN-γ in children with FA. **(B)** IL-17A was correlated with IFN-γ in healthy children. **(C)** IL-17A was correlated with IFN-γ in all participants. *P<0.05.

As shown in **Table 4** and **Figure 3**, IL-17A was positively correlated with IFN-γ in children with FAs (r=0.79) and in healthy children (r=0.98) at p < 0.05.

**Table 4:** Correlation between IL-17 and IFN-γ.

	n	r	p
FA group	20	0.79	<0.05
Healthy	20	0.98	<0.05
Total	40	0.86	<0.05

## 4 Discussion

The present study compared the levels of IL-17A and IFN-γ in the saliva of children with FAs and healthy children using an age- and sex-matched design, and the levels of salivary IL-17A and IFN-γ were higher in the FA group than in the healthy control group.

IL-17A belongs to the IL-17 family, which includes structurally similar cytokines named A to F. IL-17A plays an important regulatory role in the immune response of the body (32). Recent studies have found that in addition to Th17 cells, which secrete IL-17A, follicular helper T cells (Tfhs), specific cytotoxic T lymphocytes (CTLs), natural killer T (NKT) cells, lymphoid tissue inducer (LTi) cells, regulatory T cells (Tregs), gamma-delta (γδ) T cells, and innate lymphoid cells (ILCs) can also secrete this interleukin (32). IL-17A can induce allergen-specific Th2 cell activation and eosinophil and neutrophil accumulation (16), has synergistic effects with other cytokines, such as TNF-α, IFN-γ, andIL-1, and plays a key role in barrier immunity and allergic diseases (17, 19).A study conducted by Magdalena Żbikowska-Gotz found that during the period of obvious clinical symptoms in FA patients, the levels of IL-17A in their blood were significantly higher than those in healthy controls (33). Moreover, the relationship between serum IL-17A levels and other allergic diseases, such as asthma, allergic rhinitis (AR), and atopic dermatitis (AD), has been shown (34, 35). However, to our knowledge, no studies have reported a relationship between salivary IL-17A levels and FA.

IFN-γ is a cytokine with multiple functions, including antiviral, antitumor, and immunomodulatory functions (31). It plays a considerable role in coordinating innate and adaptive immune responses (36). FA represents a failure to induce immune tolerance, characterized by immune deviation from the Th2 cytokine pattern and delayed maturation of Th1 cells. Although IFN-γ is secreted by Th1 cells, and Paajanen L (37) found increased IFN-γ secretion in the duodenum of children with delayed-type cow’s milk allergy (CMA), which was consistent with a study by Veres et al. (38), in which children with untreated delayed-type CMA and/or cereal grain allergies had elevated densities of IFN-γ cells and a high expression level of IFN-γ mRNA in the duodenum. In our study, we found that the level of IFN-γ in the saliva of children with FA was higher than that of healthy children. This finding led us to investigate why the levels of IFN-γ were higher in FA colon tissue and saliva than in healthy tissue, which is different from the current theoretical reasoning regarding FAs. One possibility may be related to the dual effects of IFN-γ; although IFN-γ can activate immune responses to eliminate viruses in inflammatory environments, it can also prevent immune overreaction and tissue damage (39, 40). The relationship of IFN-γ with FA and its role in FA need to be further studied.

Another possible explanation is related to Th17/Th1 cells. To our knowledge, there is currently no research on the relationship between Th17/Th1 cells and FA. However, Th17 cells are abundant in mucosal tissue (9, 41) and play an important role in coordinating mucosal inflammation and tolerance as well as balancing mucosal repair and healing (9, 41). Moreover, an increasing number of studies have found that Th17 cells have high variability and can transform into Th17/Th1 cells based on the surrounding inflammatory environment (9, 41). Th17/Th1 cells can secrete both IL-17A and IFN-γ as potentially pathogenic Th17 cells (41, 42). An increasing number of studies have revealed the clinical relevance of Th17/Th1 cells and IL-17A and IFN-γ in patients with immune diseases such as autoimmune diseases (43, 44) and many other intestinal immune diseases (12–15). Given the results of the present study, whether the elevated levels of IFN-γ and IL-17A in saliva are related to Th17/Th1 cells and whether Th17/Th1 cells contribute to FA require further study.

The first limitation of our study was that because of the sex- and age-matched case–control design, it was difficult to recruit eligible participants, leading to a small sample size and fewer pairs at different ages, which may be result in significant differences between different pairs. Second, of the 20 FA children included in this study, two children were allergic to fish and shrimp, three children were allergic to both eggs and milk, and the remaining children were allergic to eggs. Therefore, it is necessary to increase the sample size to explore the relationship between other types of allergies and IFN-γ and IL-17A levels in saliva. Although the result of the general linear model showed that the observed power was high, further study is needed to explore the alteration of salivary IL-17A and IFN-γ levels in patients with FA. Moreover, the present study used a case–control design and determined only that the salivary levels of IL-17A and IFN-γ were higher in children with FAs than in healthy children. Further study is necessary regarding the important potential roles of these two cytokines in the diagnosis and treatment of FA.

To our knowledge, there is currently a limited amount of research on the relationship between salivary IL-17A and IFN-γ in patients with FA diseases. Cytokines are the main signaling molecules that coordinate the functions of numerous immune cells and produce overall effects. Cytokines usually act on nearby target cells in autocrine or paracrine manners, so cytokines mainly exert their effects locally. IL-17A and IFN-γ can act on many cytokines and play an important role in the occurrence and development of allergic diseases. The oral cavity is the starting point of the gastrointestinal immune system, and saliva can serve as a crucial source of information regarding the gastrointestinal immune environment. Therefore, our study examined the relationship of salivary IL-17A and IFN-γ levels with FA in children using a sex- and age-matched case–control design and found that the levels of salivary IL-17A and IFN-γ were higher in the FA group than in the healthy control group. The results of the present study may provide a foundation for research on new methods of diagnosing FA and evaluating the effectiveness of treatment and suggest new areas of research on the mechanism of FA.

## Data availability statement

The original contributions presented in the study are included in the article/supplementary material. Further inquiries can be directed to the corresponding authors.

## Ethics statement

The studies involving humans were approved by the Ethics Committee of the Capital Institute of Pediatrics (Approval No. SHERLLM2022017). The studies were conducted in accordance with the local legislation and institutional requirements. Written informed consent for participation in this study was provided by the participants’ legal guardians/next of kin. Written informed consent was obtained from the individual(s) and/or minor(s)’ legal guardian/next of kin for the publication of any potentially identifiable images or data included in this article.

## Author contributions

All authors meet the authorship requirements. Conception and design: YY, SO, QL, TZ, CL and YG. Development of methodology: YY, SO and WW. Data collection (collecting and managing patients): YY, YD, SX, SW, YG and CL. Data analysis and interpretation (such as statistical analysis and computational analysis): YY, SO and MZ. Writing, review, and/or revision of the manuscript: YY, SO, QL, YD, SQ, MZ, TZ, and YG. Research supervision and guidance: TZ, YG, and CL. All authors contributed to the article and approved the submitted version.
